# A Comprehensive Characterization of Genome-Wide Copy Number Aberrations in Colorectal Cancer Reveals Novel Oncogenes and Patterns of Alterations

**DOI:** 10.1371/journal.pone.0042001

**Published:** 2012-07-31

**Authors:** Tao Xie, Giovanni d’ Ario, John R. Lamb, Eric Martin, Kai Wang, Sabine Tejpar, Mauro Delorenzi, Fred T. Bosman, Arnaud D. Roth, Pu Yan, Stephanie Bougel, Antonio Fabio Di Narzo, Vlad Popovici, Eva Budinská, Mao Mao, Scott L. Weinrich, Paul A. Rejto, J. Graeme Hodgson

**Affiliations:** 1 Oncology Research, Pfizer Worldwide Research and Development, San Diego, California, United States of America; 2 Swiss Institute of Bioinformatics, Lausanne, Switzerland; 3 University Hospital Gasthuisberg, Katholieke Universiteit Leuven, Leuven, Belgium; 4 Lausanne University Medical Center, Lausanne, Switzerland; 5 Geneva University Hospital, Geneva, Switzerland; Pontificia Universidad Catolica de Chile, Chile

## Abstract

To develop a comprehensive overview of copy number aberrations (CNAs) in stage-II/III colorectal cancer (CRC), we characterized 302 tumors from the PETACC-3 clinical trial. Microsatellite-stable (MSS) samples (n = 269) had 66 minimal common CNA regions, with frequent gains on 20 q (72.5%), 7 (41.8%), 8 q (33.1%) and 13 q (51.0%) and losses on 18 (58.6%), 4 q (26%) and 21 q (21.6%). MSS tumors have significantly more CNAs than microsatellite-instable (MSI) tumors: within the MSI tumors a novel deletion of the tumor suppressor WWOX at 16 q23.1 was identified (p<0.01). Focal aberrations identified by the GISTIC method confirmed amplifications of oncogenes including EGFR, ERBB2, CCND1, MET, and MYC, and deletions of tumor suppressors including TP53, APC, and SMAD4, and gene expression was highly concordant with copy number aberration for these genes. Novel amplicons included putative oncogenes such as WNK1 and HNF4A, which also showed high concordance between copy number and expression. Survival analysis associated a specific patient segment featured by chromosome 20 q gains to an improved overall survival, which might be due to higher expression of genes such as EEF1B2 and PTK6. The CNA clustering also grouped tumors characterized by a poor prognosis BRAF-mutant-like signature derived from mRNA data from this cohort. We further revealed non-random correlation between CNAs among unlinked loci, including positive correlation between 20 q gain and 8 q gain, and 20 q gain and chromosome 18 loss, consistent with co-selection of these CNAs. These results reinforce the non-random nature of somatic CNAs in stage-II/III CRC and highlight loci and genes that may play an important role in driving the development and outcome of this disease.

## Introduction

Colorectal cancer (CRC) ranks second to lung cancer in both incidence and mortality in developed countries [1]. It is characterized by highly complex patterns of somatic genetic alterations of oncogenes and tumor suppressors that drive initiation and progression [2], [3], [4]. Understanding the cellular and molecular mechanisms by which these genetic changes facilitate colon cancer formation is critical for development of targeted therapeutic strategies aimed at controlling disease progression while minimizing toxic side effects.

One well-established genetic mechanism by which cancer cells alter the activity of oncogenes and tumor suppressors is through changes in gene dosage. Detailed characterization of DNA copy number aberrations (CNAs) have helped identify important oncogenes including ERBB2 and EGFR, as well as tumor suppressors such as TP53 [5]. Numerous studies have documented genome-wide somatic CNAs in CRC [6], [7], [8], [9], [10], [11], [12], [13], [14], [15], [16], [17], [18], some of which have been linked to clinical outcome or metastatic progression [19], [20], [21], [22], [23], [24]. However, many of these studies have been limited by modest sample size, low resolution assays, or lack of associated clinical annotation, particularly for early-stage (II/III) colon cancer. Consequently, a comprehensive overview of CNAs and their association with outcome in stage II/III colon cancer has not been developed.

We surveyed somatic CNAs in a collection of 302 stage II/III colon cancers derived from the Pan-European Trials in Adjuvant Colon Cancer (PETACC)-3 trial, a large randomized phase III assessment of the role of irinotecan added to fluorouracil (FU)/leucovorin (FA) as adjuvant treatment for colon cancer [25]. The results presented herein explore the relationship between CNA, mRNA [26] and outcome, and contribute to a comprehensive molecular overview of stage-II/III colon cancer, which is paramount for refining patient classification and effective treatment.

## Materials and Methods

### Clinical and mRNA Data for PETACC-3 Patients

All stage II/III colon cancer patients included in this study were derived from the PETACC-3 clinical trial [25], with at least 5 years of clinical follow-up for each patient. The age, gender, stage, MSI (microsatellite-instable) as well as BRAF and KRAS mutation status of the patient population are listed in **Table S1**. mRNA expression data was generated on the ALMAC Colorectal Cancer DSA platform (Craigavon, Northern Ireland), as reported previously [26]. Patient and ethics approval for this study was obtained from the PETACC-3 Translational Research Working Party (PTRW).

### Molecular Inversion Probe Data Generation

DNA extractions were performed on macrodissected formalin-fixed, paraffin-embedded (FFPE) tumor tissue derived from a single 5 uM slide from 835 patient samples. Tumor tissue within each section was identified and labeled by a qualified pathologist (F. Bosman). For normal controls, DNA was extracted from samples with sufficient amounts of histopathologically normal adjacent tissue well away from the tumor margins. DNA was quantified using the picogreen assay. For samples that yielded less than the recommended input DNA amount (75 ng), all DNA was carried forward into the Molecular Inversion Probe (MIP) amplification, labelling, and hybridization protocols using Affymetrix’s OncoScan V1.0 FFPE Express services (Affymetrix, CA). Samples that failed PCR amplification or displayed a Median Average Pairwise Difference (MAPD) >0.6 after hybridization were removed from the final analysis, resulting in 302 tumor samples along with 44 adjacent normal samples as the normal baseline comparator. Typically samples below 20 ng of input DNA failed the MIP amplification cutoff and were not carried forward to array hybridization. Samples with at least 75 ng of input DNA universally yielded high quality copy number data (MAPD<0.6). Results varied for input DNA amounts of 20–75 ng, where the MAPD>0.6 filter served to eliminate excessively noisy samples.

### Copy Number Data Analysis

Copy number data was analyzed with the Nexus Copy Number 6.0 software (Biodiscovery, Inc., CA, USA). The raw copy number data for each probe provided by Affymetrix was smoothed by a quadratic correction provided by NEXUS and centered using diploid regions. CNA frequency comparisons amongst sample groups (e.g. MSS versus MSI; stage-II versus stage-III) was performed using NEXUS default thresholds of >15% difference and significance p<0.01 (Fisher’s exact test). To generate copy number segments and minimal common regions (MCRs), we applied a modified version of the Circular Binary Segmentation (CBS) algorithm [27] called “Rank Segmentation” in NEXUS. The p-value cutoff for CBS was 1.0E–6, and segments were assigned to 1 of 5 bins: amplified (>3.8 copies), gained (2.3 to 3.8 copies), unchanged (1.7 to 2.3 copies), deleted (0.5 to 1.7 copies) or homozygously deleted (<0.5 copies). For MCR frequency significance testing, we used a p-value cutoff of <0.01 from the statistical Significance Testing for Aberrant Copy number (STAC) method [28]. Hierarchical clustering of CNA was performed in NEXUS too (complete linkage, sex chromosomes ignored). To detect focal amplifications, we applied GISTIC (Genomic Identification of Significant Targets in Cancer) version 2.0 [29] using a Q-value cutoff <0.25. Genes reported in GISTIC2 amplification peaks were further examined if they are enriched in any biological pathways. We used canonical pathway database provided by MSigDB [30]. Pathway gene sets with less than 10 members or greater than 500 members were excluded. Fisher’s exact test was used to access if those genes are over-represented. FDR was calculated based on 100 permutations where random sets of genes of same size were tested. We also used Fisher’s exact test to see if frequencies of certain CNAs differ among patient groups (stage II vs. III, MSI vs. MSS etc). Survival analysis was performed using the Kaplan–Meier method with a p value (log-rank test) cutoff of <0.01. For analysis of CNA/CNA correlations, the Pearson correlation was computed at the gene level for all pairs of genes as described previously [31]. To derive gene level summaries from the copy number data, we assigned the copy number values from the segment(s) overlapping each gene: when there were multiple segments within the gene boundary, we averaged the copy numbers from those segments. All genome-based data reported in this manuscript are based on NCBI build 36 (hg18) of the human genome.

### Expression Data Analysis

Gene expression data from the PETACC-3 patients was reported previously [26]. We matched it with gene level copy number data by ENTREZ ID. Copy number and gene expression data were simultaneously available for 213 of the 269 MSS patients with available CNA data. To test cis-correlation between a gene’s copy number and its own mRNA expression level across tumors, we categorized patients according to their aberration status (amplification, gain, no-change, loss or homozygous deletion) associated to the expression values of probe sets mapping to the same gene.

## Results

### Copy Number Aberrations and Microsatellite Instability

33 of the 302 samples in our analysis were microsatellite instable (MSI): consistent with previous studies [19], [32], the average number of CNAs in MSI tumors (10.2±6.5) was significantly smaller (p<0.01, two sample t-test) than the average number of CNAs in microsatellite stable (MSS) tumors (33.2±17.6). Nevertheless, two focal regions were deleted significantly more frequently in MSI samples: chr16q23.1 (chr16∶77,231,391–77,261,567 bp) in 24.2% of MSI samples vs. 7.1% of MSS samples (p<0.01), and chr20q11.1 (chr20∶28,118,678–28,244,164) in 24.4% of MSI samples vs. 8.9% in MSS samples (p<0.01). Interestingly, the only gene contained within the 16 q23.1 locus is the WWOX tumor suppressor, an inhibitor of the WNT/beta-catenin pathway [33], which is frequently activated in colon cancer.

### Recurrent CNAs, Novel Oncogenes and Affected Pathways

**Figure 1:**
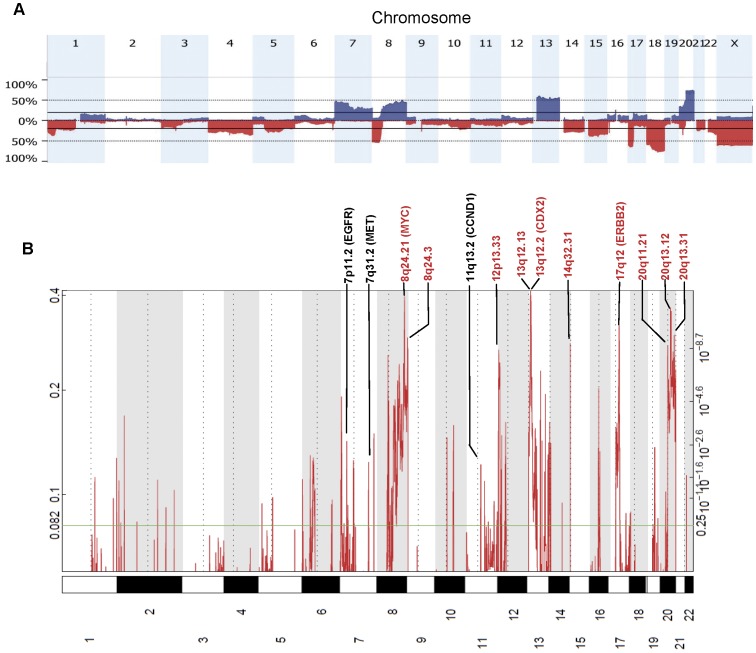
Summary of copy number aberrations detected in 269 MSS stage II/III colon cancer samples. (**A**) Frequencies of copy number gain (above axis, blue) and copy number loss (below axis, red) across the human genome. (**B**) Significance of focal amplifications detected by GISTIC 2.0. Chromosome positions were indicated along the y axis with centromere positions indicated by dotted lines. The ten most significant GISTIC peaks are shown in red text. Additional GISTIC peaks encoding established oncogenes are in black text. Details for all GISTIC peaks are provided in Table S3.

**Figure 2:**
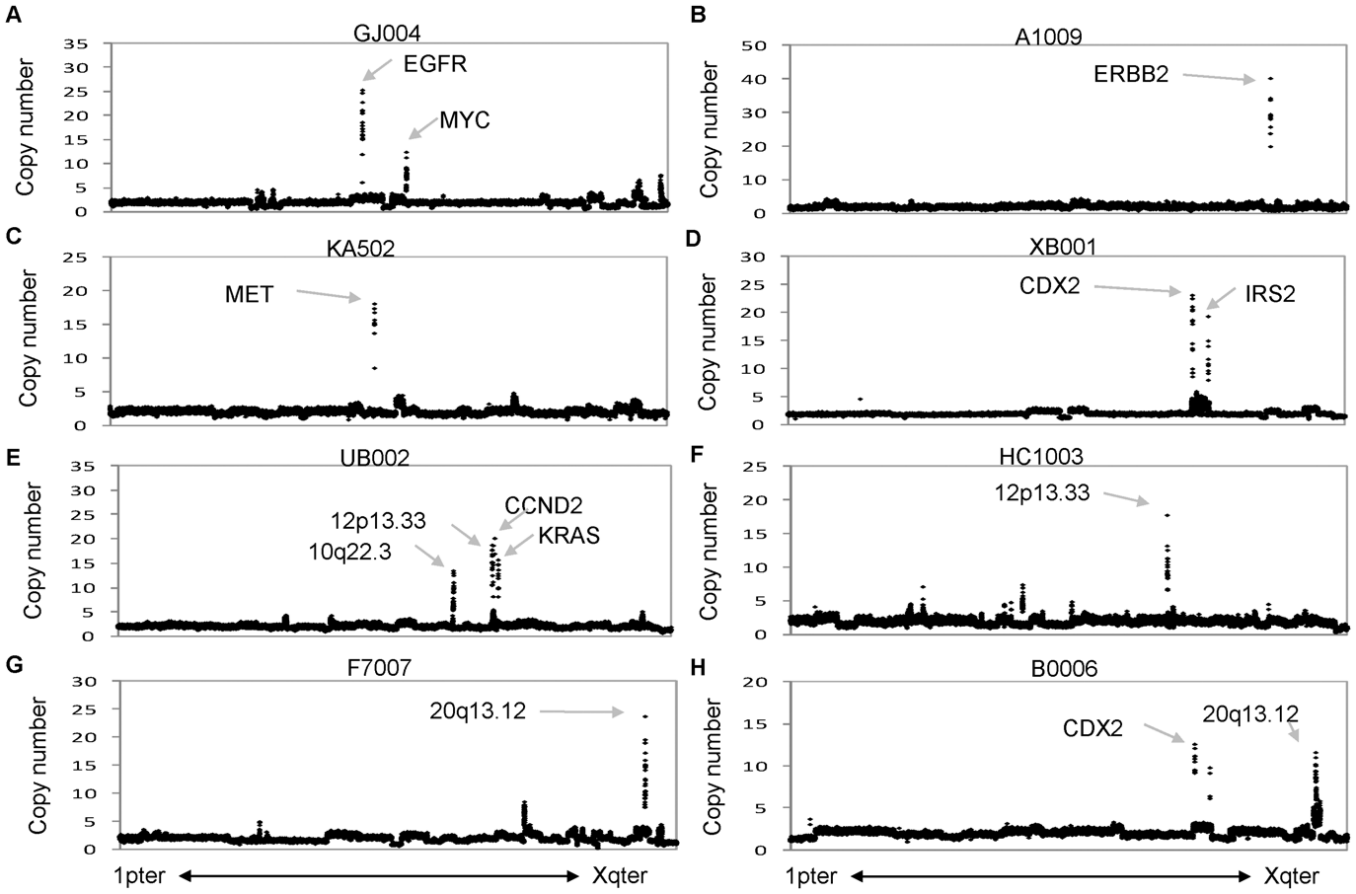
Focal amplification of genomic loci in selected stage II/III colon cancer samples. (**A–H**) Copy number plots for the entire genome arranged in chromosomal order from the short arm of chromosome 1 (1pter) to the long arm of chromosome X (Xqter) for 8 independent tumor samples. Amplicons of particular interest are highlighted with arrows, along with established oncogenes. Details regarding all amplicons and GISTIC peaks are in Table S3.

Given the relatively low CNA prevalence in MSI tumors, we focused our analyses on the 269 MSS tumors. As has been reported previously [7], [8], [9], [10], [11], [12], [13], [14], [15], [16], [17], the frequencies of copy number gains and losses across the genome were not randomly distributed (**Figure 1A**), with CNAs ranging from single copy gains and losses of broad chromosomal regions, to focal homozygous deletions and high-level amplifications (**Figure 2**). The most frequent regions of gain encompassed chromosomal regions 7 p, 8 q, 13 q, and 20 q, and the most frequent regions of loss encompassed 8 p, 17 p, and 18 q (**Figure 1A**).

**Figure 3:**
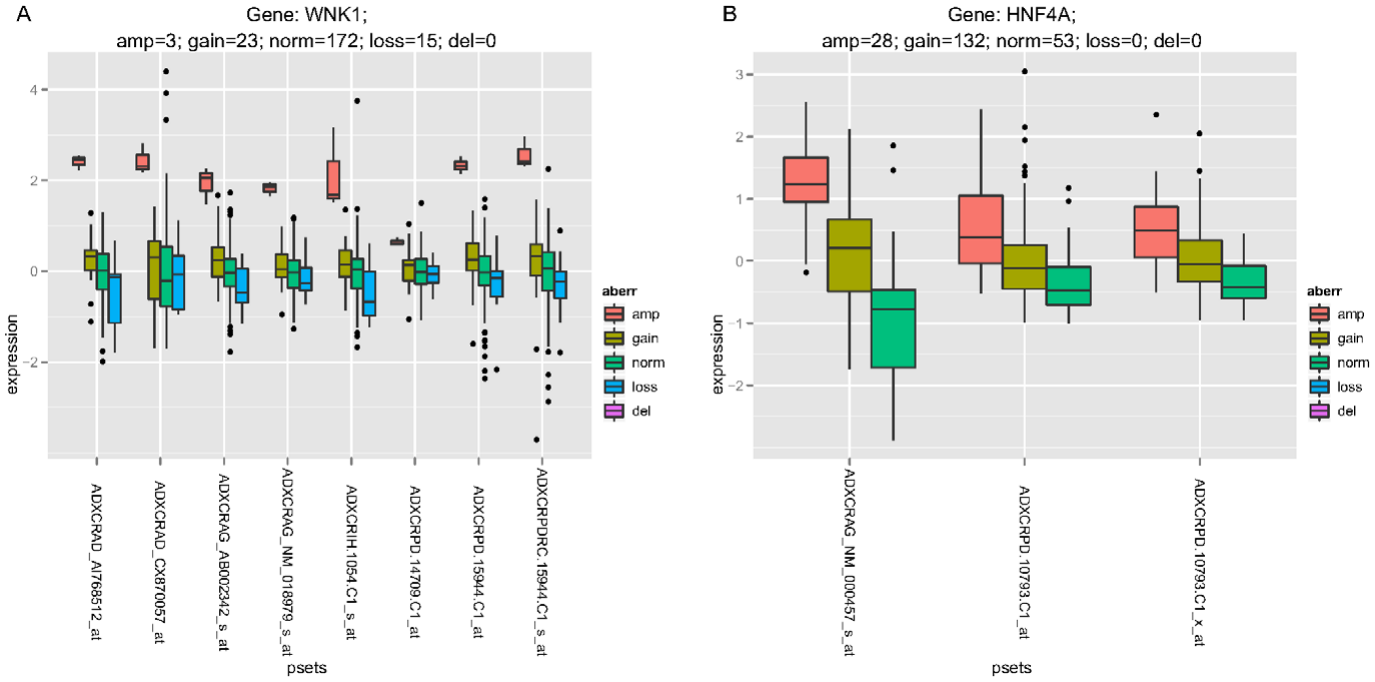
Boxplots for WNK1 (A) and HNF4A’s (B) mRNA expression grouped by CNA status. Tumor samples were categorized by their CNA status (deletion, loss, normal, gain, amplification) for the indicated gene. The panels show the expression level by category for each probeset from the ALMAC platform (see Materials and Methods) representing the specific gene. The values were centered for each probeset; categories are plotted if there was at least one sample in it.

To gain further insight, we summarized recurrent chromosomal gains and losses into Minimal Common Regions (MCRs) using Significant Testing of Aberrant Copy Number (STAC) [28], and GISTIC [29] to highlight candidate oncogenes in the MCRs based on the focality and amplitude of copy number change. A total of 66 MCRs were identified at frequencies above 10% (**Table S2**): there were 25 MCRs of gain ranging from 251 Kb to 104 Mb, and 41 MCRs of loss ranging from 286 kb to 138 Mb. GISTIC helped to refine the MCRs to loci and genes of particular significance (**Table S3**)**.** Many of the significant peaks identified by GISTIC contained established oncogenes including CCND1, CDX2, EGFR, ERBB2, MET, and MYC (**Figure 1B**), along with tumor suppressors such as APC, SMAD4, and TP53. Several of the oncogenic peaks were driven by high-amplitude focal events in a subset of tumors (**Figure 2**), and these focal amplifications led to significant increases in mRNA expression for several of these genes. Highly significant GISTIC peaks not associated with well-established oncogenes or tumor suppressors include 12 p13.33 (**Figure 2E, F**) and 20 q13.12 (**Figure 2G, H**), which had recurrent high-magnitude focal amplifications, as well as 14 q32.31 which, although not highly amplified, had gains of sufficient recurrence and focality as to render a highly significant GISTIC Q-value (**Figure 1B**, **Table S3**). With the GISTIC amplicon data, we summarize 114 candidate cancer drivers in **Table S4**, which include twelve (10%) established oncogenes such as MYC, KRAS, and MET. Putative oncogenes including WNK1 (**Figure 3A**) and HNF4A (**Figure 3B**) have Q-score, amplified frequency, and cis-acting effects on mRNA that are comparable to established oncogenes **(Figure S1).** Our analysis has narrowed more than 6,000 genes from MCR regions of the genome to a manageable number of about 100 for further experimental validation.

To further search for patterns of affected pathway alterations, we mapped the list of genes amplified in CRC (**Table S4**) onto canonical molecular signaling pathways and cellular processes. **Table 1** shows top canonical pathways possibly affected by the amplified genes. Cell cycle is one of the most enriched pathways affected by somatic CNA involving genes such as CCND1, MYC, TFDP1 and YWHAZ. KEGG “Pathways in Cancer” underlies the broad spectrum effect of somatic CNAs in targeting multiple key pathways in cancer simultaneously. More specifically, we also identified individual cancer-related pathways that are significantly over-represented among cis-acting genes driven by somatic CNAs, including ERBB signaling pathway and MAPK kinase signaling pathway. Taken together, these results suggest that these somatic CNAs encode novel oncogenic driver genes and potential therapeutic targets in colon cancer.

**Table 1:** Top canonical pathways possibly affected by the amplified genes.

Term	P-value	FDR*	Fold enrichment	% tumor amplified
KEGG_ADHERENS_JUNCTION	1.79E-04	4.50E-03	14.37	11.2%
KEGG_CELL_CYCLE	1.35E-03	2.01E-02	8.42	10.4%
KEGG_PATHWAYS_IN_CANCER	1.44E-03	2.32E-02	4.93	9.7%
KEGG_ERBB_SIGNALING_PATHWAY	3.16E-04	5.83E-03	12.39	8.2%
SIG_PIP3_SIGNALING_IN_CARDIAC_MYOCTES	1.68E-03	2.42E-02	12.83	6.3%
BIOCARTA_TEL_PATHWAY	3.90E-05	2.13E-03	44.90	5.6%
KEGG_AXON_GUIDANCE	1.24E-02	7.48E-02	6.27	5.6%
KEGG_MAPK_SIGNALING_PATHWAY	1.77E-02	8.82E-02	4.04	5.6%

* FDR is based on was calculated based on 100 permutations where random sets of genes of same size were tested.

### CNA Clustering and Non-random CNA Correlations in CRC

**Figure 4:**
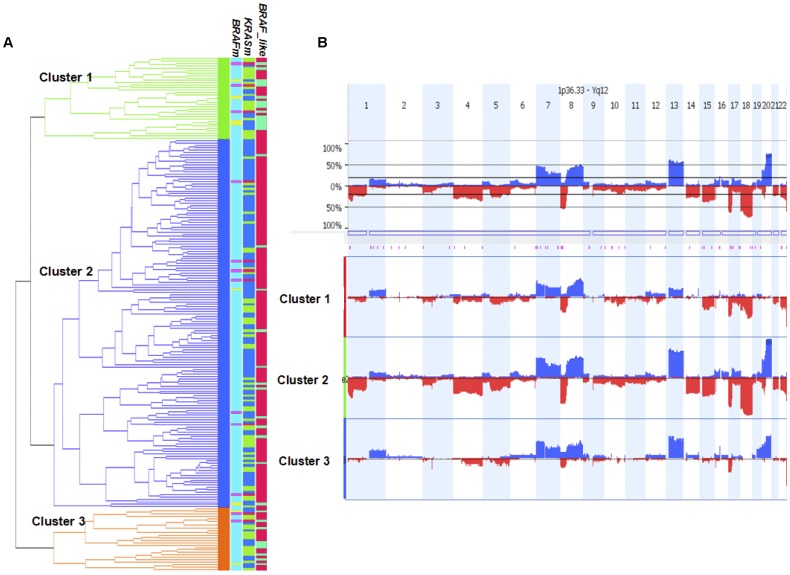
Unsupervised hierarchical clustering analysis based of genome-wide copy number data. (**A**) Three major clusters. The right-hand annotation indicates, in order, the BRAFm (in yellow, BRAF mutants; in blue, BRAF wild-types), KRASm (mutants in green), and BRAFm-like (in green, BRAFm-like; in red, non-BRAFm-like). Purple color indicates missing values. (**B**) Genome-wide frequency plot of copy number gain (above axis, blue) and copy number loss (below axis, red) across three major clusters.

We performed unsupervised hierarchical clustering of the global CNA data and identified three major clusters. Though we didn’t find significant associations to age, gender, stage or KRAS mutation status, we observed that BRAF wild type tumors were significantly enriched in the largest cluster and BRAF mutants in one of the smaller clusters (p<0.01). Previously we [26] developed a BRAF-mutant gene expression signature from the PETACC-3 cohort and studied its prognostic implications. Among 213 MSS patients with mRNA expression data available, the signature identified 37 “BRAFm-like” samples (including 8 BRAF mutants) as well as 176 “non-BRAFm-like” samples. We re-ran clustering analysis on those 213 samples (**Figure 4A**), and found very significant enrichment of “non-BRAFm-like” samples (p<0.01) in the largest cluster (cluster 2) and “BRAFm-like” samples in cluster 1 (P<0.01, **Table 2**). Compared to cluster 2, cluster 1 shows much lower frequencies of amplification/deletion events, especially on chr13 q, 14 q, 18 q and 20 q (**Figure 4B** **)**. A closer look reveals that cluster 1 is completely depleted from CNAs at chr20 while 95% of cluster 2 samples had chr20 amplified. These results corroborate with the observation of relative lower expression of chr20 genes in BRAFm-like with respect to the rest of the BRAFwt samples [26].

**Table 2:** Unsupervised hierarchical clustering indentified three major CNA clusters.

Cluster	samples	BRAFm-like	non-BRAF-like	BRAFm	BRAFwt	missing
**1**	34	16*	18	4*	27	3
**2**	153	12	141*	2	144*	7
**3**	26	9	17	2	22	2
**Subtotal**	213	37	176	8	193	12

* indicates significant over-representation in the category.

**Figure 5:**
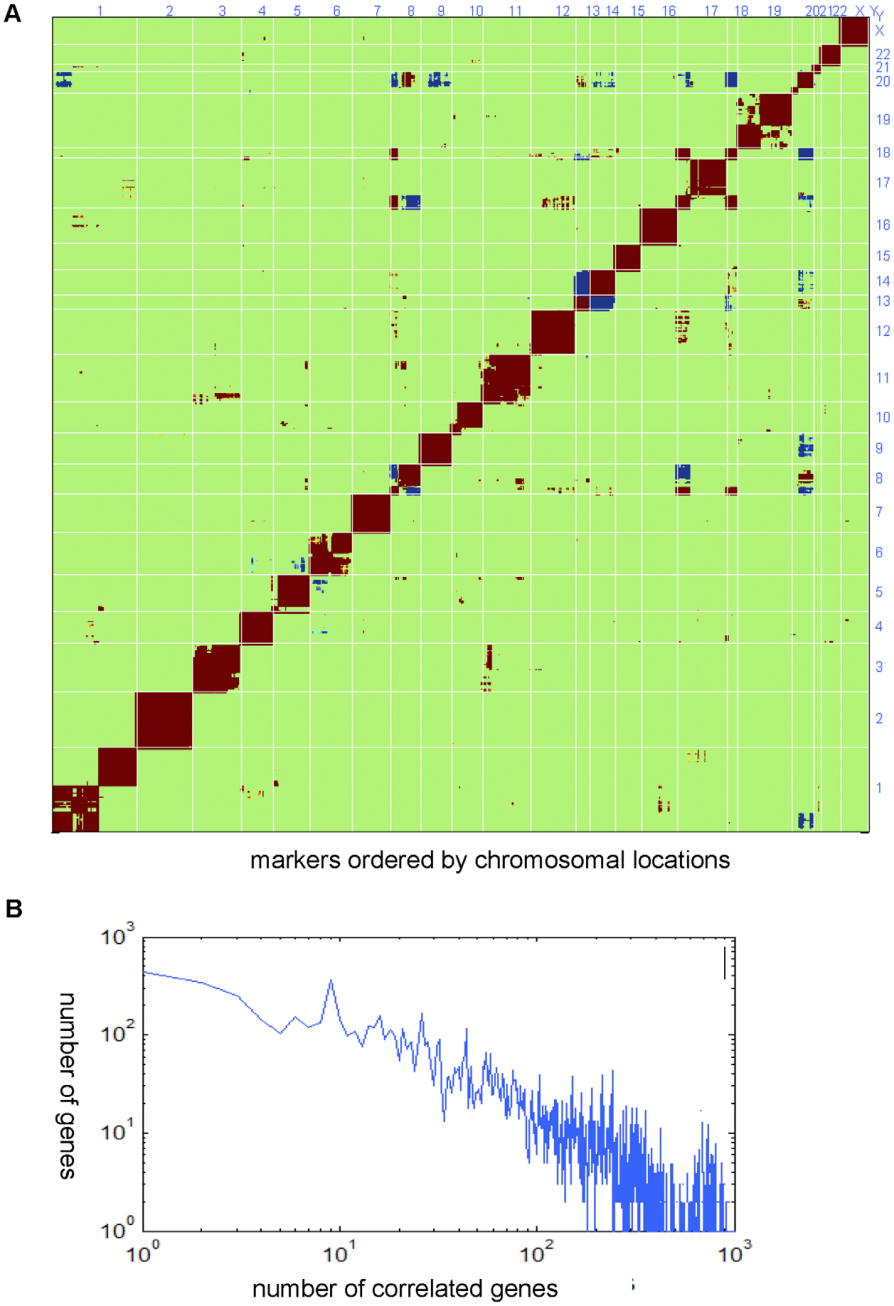
Pair-wise DNA/DNA correlations reveal significant associations between unlinked loci. (**A**) Pair-wise correlations computed from gene copy number are ordered by chromosomal positions through the genome on the X and Y axes, with red indicating a positive correlation and blue indicating a negative correlation. The red diagonal represents the correlation of a gene with itself. The lower right and upper left portions of the graph represent mirror images of each other showing the copy number correlations of unlinked loci. (**B**) Log/log plots for significant gene/gene correlations (|R|≥0.3).

We previously reported that in cell lines CNAs at unlinked loci were frequently correlated to each other and that such correlations were likely the result of selection [31]. To assess whether a similar phenomenon was evident in clinical stage II/III MSS colon cancer, we conducted pair-wise correlations of copy number for all genes (∼22 k) across the genome. As expected, adjacent (linked) genes were highly correlated (**Figure 5A**, close to diagonal). At a higher level some chromosome arms became unlinked (e.g. chr1p vs. 1 q, 10p vs. 10 q) or anti-correlated (e.g. chr8 p vs. 8 q). In addition, there were numerous correlations between unlinked loci (**Figure 5A**, off-diagonal), suggesting co-selection of these genomic regions. For example, chromosome 8 p losses were correlated to losses of chromosomes 17 p and 18, along with gain of chromosome 20 q. Chromosome 13 gains were correlated to chromosome 14 losses. The distribution of gene-gene associations was significantly different than a randomization of the CNV data (**Figure 5B**). Similar to what was found in other cancer settings [31], [34] there was a scale-free structure where a few genes were highly correlated to many other genes, while most genes correlated to only a few genes. This suggests that a small number of DNA loci act as hubs in a highly nonrandom hierarchical structure.

### Relationship of CNA to Stage and Outcome

To identify individual CNAs that associate with tumor stage, we compared CNA frequencies between stage II (n = 30) and stage III MSS samples (n = 239). While both groups had similar patterns of CNA, a deletion on chromosome 3p14.2 had significantly (p<0.01) higher frequency in stage III tumors (24.3%) compared to stage II tumors (3.3%). This locus encodes FHIT, a candidate tumor suppressor and apoptotic regulator in colorectal cancer [35], and the higher frequency of deletion in stage III tumors suggests that loss of FHIT function may contribute to the progression of colon cancer from a lower to higher stage disease.

The large set of stage II/III MSS colon cancer samples with associated time-to-relapse, recurrence-free-survival (RFS) and overall survival (OS) afforded a unique opportunity to identify CNAs associated with outcome. Using Kaplan-Meier analysis, we first investigated whether the ch20q amplification revealed by sample clustering described previously lead to statistically significant differences in survival probability. A gained MCR on chromosome 20 q11.21-q13.33 (chr20∶29,297,270–62,435,964 bp) was significantly associated with improved OS in stage III tumors (p<0.01). GISTIC identified one amplicon in this MCR on 20q13.33 (chr20∶61,440,621–61,778,204 bp) which was also significantly associated with improved OS. This region of approximately 300 kb contains one interesting genes such as EEF1A2 and PTK6. Anand et al. reported [36] EEF1A2’s over-expression in about 30% of ovarian tumors and some established ovarian cancer cells. However, high EEF1A2 protein expression was associated with significantly increased 20-year survival probability in women with serous ovarian tumors [37], or in primary breast tumors, and this protective effect is thought to be due to EEF1A2’s high expression in reducing the aggressiveness [38]. PTK6 was also reported [39] as positive associated to metastases-free survival in breast cancer; and shows strong cis CN/mRNA correlation in our analysis (**Table S4**). Here the CNA data suggest that amplification of the 20 q13.33 locus could be a significant prognostic marker of CRC cancer.

Besides chr20q amplification, we applied Kaplan-Meier analysis to assess the relationship of all other MCRs and GISTIC peaks with RFS and OS. There were no significant associations between MCRs or GISTIC peaks versus OS or RFS for stage II tumors, possibly reflecting the limited number of samples in this group (n = 30). However, a deletion on chromosome 10 p (Chr10∶0–10,743,764 bp) was significantly associated with poor RFS in stage III tumors alone (p<0.01) or stage II/III tumors combined (p<0.01), as well as poor OS in stage II/III tumors combined (p<0.01). Similarly, a deleted MCR on 19 p13.12 (chr19∶14,425,490–15,580,441 bp) was significantly associated with OS (p<0.01) in stage II/III tumors combined (**Figure S2**).

## Discussion

The main goals of this study were to develop a comprehensive overview of copy number aberrations (CNAs) and their associated genes in stage II/III colon cancer, to elucidate the underlying biology, and to associate CNAs with outcome. Regions of recurrent and focal CNA identified in these tumors highlight genomic regions most likely to encode oncogenes and tumor suppressors. Established oncogenes identified in this study that represent positive controls include MYC, CDX2, EGFR, MET, ERBB2, and CCND1.

The most prominent novel amplicons identified in this study include 12 p13.33 and multiple loci on 20 q (20 q11.21, 20 q13.12, 20 q13.31). The 12 p13.33 amplicon encodes the intriguing candidate WNK1, a member of the WNK family of serine/threonine kinases which affect MAPK signaling and a variety of cancer hallmarks including cell cycle progression, evasion of apoptosis, invasion and metastasis, and metabolic adaptation [40]. The complex pattern of gains and amplification on chromosome 20 q suggest multiple oncogenic drivers on this chromosome arm, consistent with observations in breast tumors [41]and other cancer types. The 20 q13.12 amplicon, which was observed in multiple tumors (**Figure 2G, 2H**) and is the most significant GISTIC peak on 20 q, encodes 11 genes, none of which have been unequivocally described as oncogenic drivers in colon cancer. Nonetheless, the reported functions of some of these genes suggest that further investigation is warranted. For example, the transcription factor HNF4A controls epithelial cell polarity and promotes gut neoplasia in mice [42]. WISP2 (WNT1 Inducible Signaling Pathway protein 2/CCN5) regulates the activity of the transforming growth factor â (TGFâ) signaling pathway and expression of genes associated with the epithelial-to-mesenchymal transition [43]. The peak at 20 q13.31 encodes BMP7, a member of the TGFâ superfamily of proteins whose overexpression in colorectal cancer significantly correlates with markers of pathological aggressiveness such as liver metastasis and is an independent prognostic factor of overall survival [44]. Functional characterization of these and other candidate oncogenes in colon cancer cell culture, patient-derived xenografts, or genetically engineered mouse models will help elucidate potential functional implications. Pathway analysis presented previously provides not only a better understanding of the possible biological context of candidate CNA drivers but also help to infer other genes on the altered pathway for which therapeutic options may be available. On the other hand, survival analysis shows improved overall survival for the sample segment with chr20 q13.33 amplification. This association contrasts with findings of another group who reported amplification of 20 q13 is indicating worse overall survival in sporadic colorectal cancers [**45**]. The exact basis for this discrepancy with our findings for is not clear, although the analyses of Aust et al. were on a substantially smaller cohort (120 samples).

Our analyses of associations between CNA and outcome in this set of stage II/III colon cancers revealed three loci that were significantly associated with overall survival (OS) or recurrence free survival (RFS). Deletion of the distal tip of chromosome 10 p (10 p15.3-p14) was associated with poor OS and RFS, while an interstitial deletion of chromosome 19 p (19 p13.12) was associated with poor OS, and gain of 20 q was associated with significantly better OS in stage III tumors. While 10 p deletions, 19 p deletions, and 20 q gains have been previously reported in stage II/III colon cancers [16], none of these loci have been previously linked to outcome in these tumors. Conversely, we did not observe significant associations of outcome to previously reported CNAs such as deletion of 16 p13.2 in stage II/III colon cancer [46], or deletion of 5 q34 and gain of 13 q22.1 in stage II tumors [17]. One potential explanation for these apparent discrepancies may relate to the limited power of the respective studies. For stage III MSS tumors, our results represent analyses of markedly higher sample numbers (n = 239) compared to published work (for e.g. 31 stage III tumors in [46]). For stage II MSS tumors, our sample set is underpowered, representing 30 samples compared to 41 [46] and 39 [17] tumors in earlier studies. These results emphasize the need for comprehensive analyses of large collections of clinically annotated tumor samples such as the stage III MSS tumor set described in this work.

We also reported here a significant non-random correlation of unlinked DNA loci with a scale-free structure in stage II/III colon cancer. These highly connected structures suggest a cycle of random changes in copy number followed by selection of a subset of changes that confer a selective advantage to tumor initiation and progression. While this is a long standing idea in cancer, correlation between unlinked loci suggests that highly ordered structures can emerge, potentially focused around biological functions of importance to the tumor. Future analyses could assess the effect of unlinked copy number correlations on gene expression, including enrichment of pathways and networks, and determining if the mRNA controlled by a pair of correlated loci overlap, where an independent effect of each loci was observable. This would identify pathways that were selectively altered during tumorigenesis and which therefore may represent new targetable functions.

## Supporting Information

Figure S1 **Boxplots for EGFR, ERBB2 and MYC’s mRNA expression grouped by CNA status.**
(PPT)

Figure S2 **Kaplan-Meier curves demonstrate CNAs showing significant association with overall survival.**
(PPT)

Table S1 **Characteristics of patients Included in the study.**
(XLS)

Table S2 **Minimal common regions identified in 269 MSS stage-II/III colon cancer samples.**
(XLS)

Table S3 **GISTIC peaks identified in 269 MSS stage-II/III colon cancer samples.**
(XLS)

Table S4 **Affected genes in selected GISTIC amplicons.**
(XLS)
