# Supplementary material for: A Comprehensive Characterization of Genome-Wide Copy Number Aberrations in Colorectal Cancer Reveals Novel Oncogenes and Patterns of Alterations
Source: PLoS One. 2012 Jul 31;7(7):e42001. doi: 10.1371/journal.pone.0042001 (PMC3409212; doi:10.1371/journal.pone.0042001)
Supplement: Figure S1 — Boxplots for EGFR, ERBB2 and MYC’s mRNA expression grouped by CNA status. (PPT) [file pone.0042001.s001.ppt]

## Slide 1
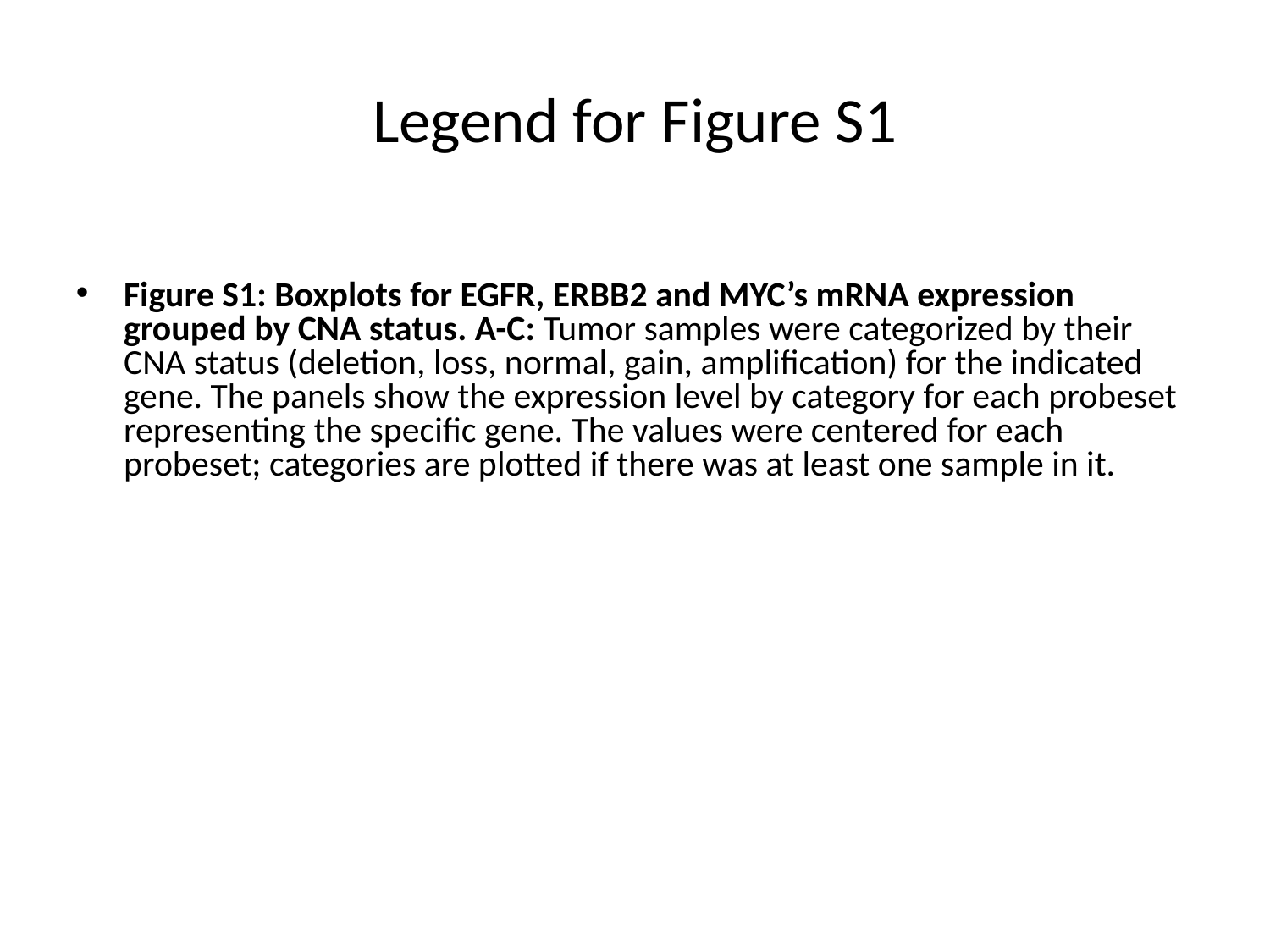

# Legend for Figure S1
Figure S1: Boxplots for EGFR, ERBB2 and MYC’s mRNA expression grouped by CNA status. A-C: Tumor samples were categorized by their CNA status (deletion, loss, normal, gain, amplification) for the indicated gene. The panels show the expression level by category for each probeset representing the specific gene. The values were centered for each probeset; categories are plotted if there was at least one sample in it.

## Slide 2
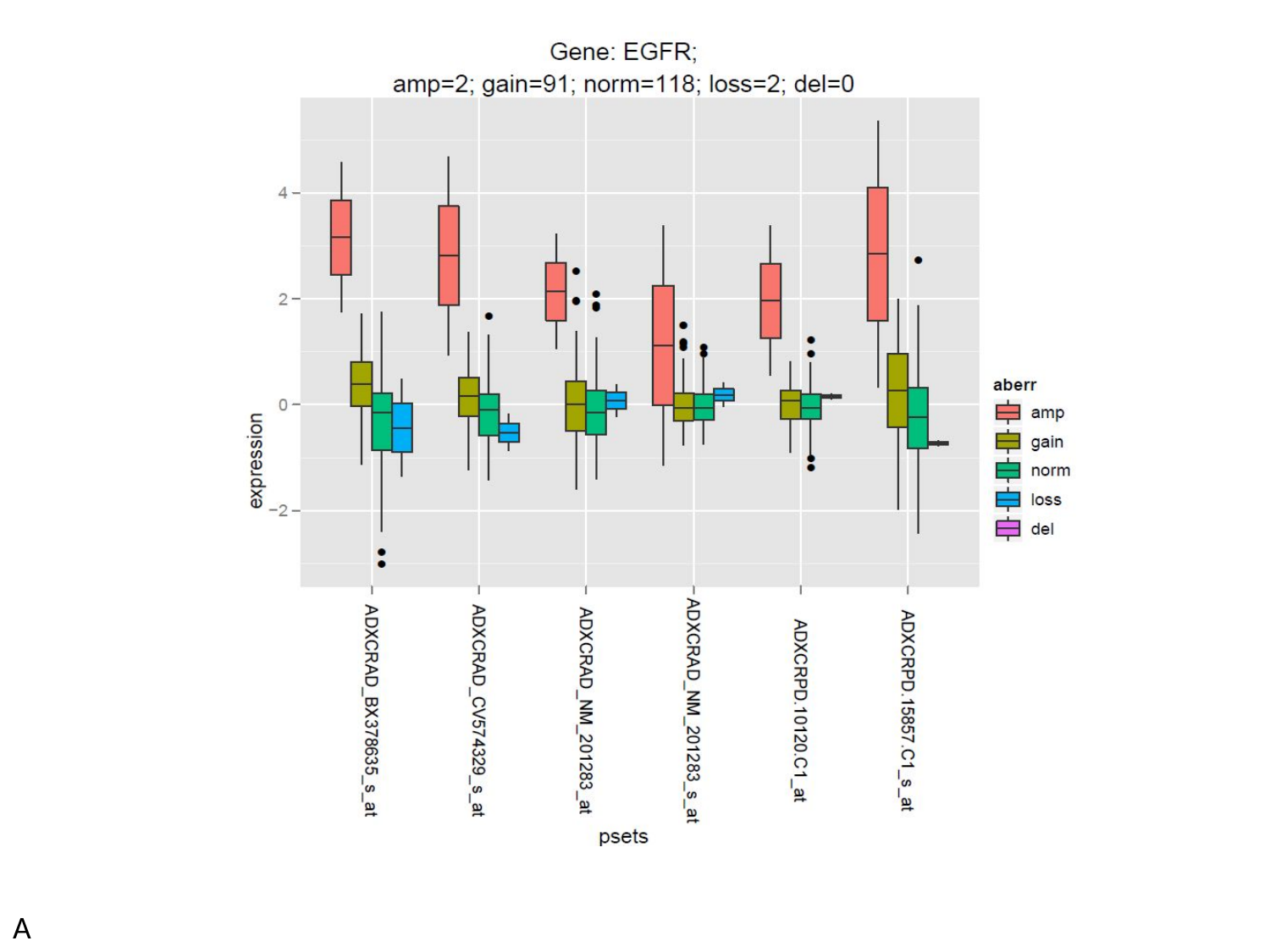

A

## Slide 3
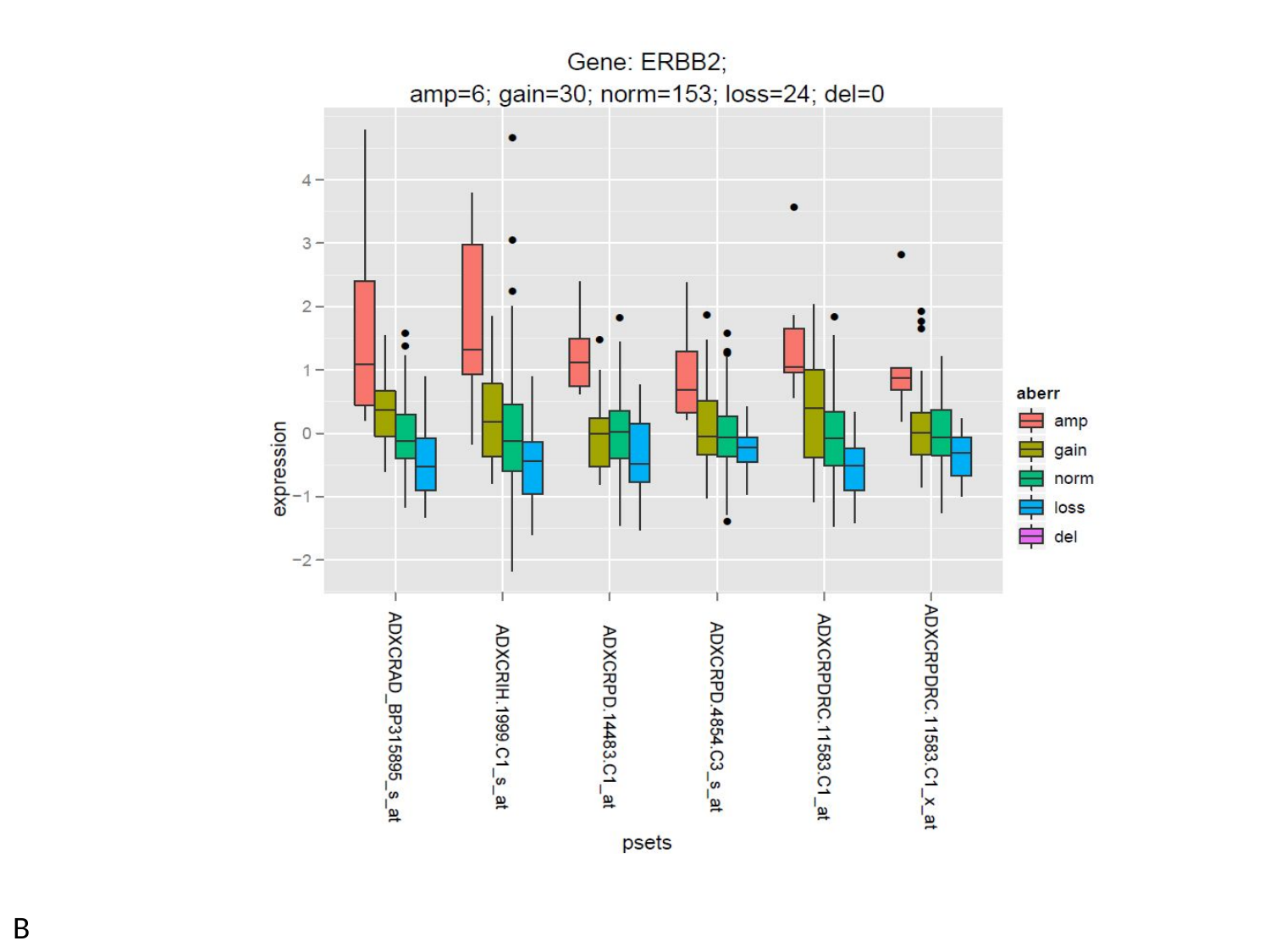

B

## Slide 4
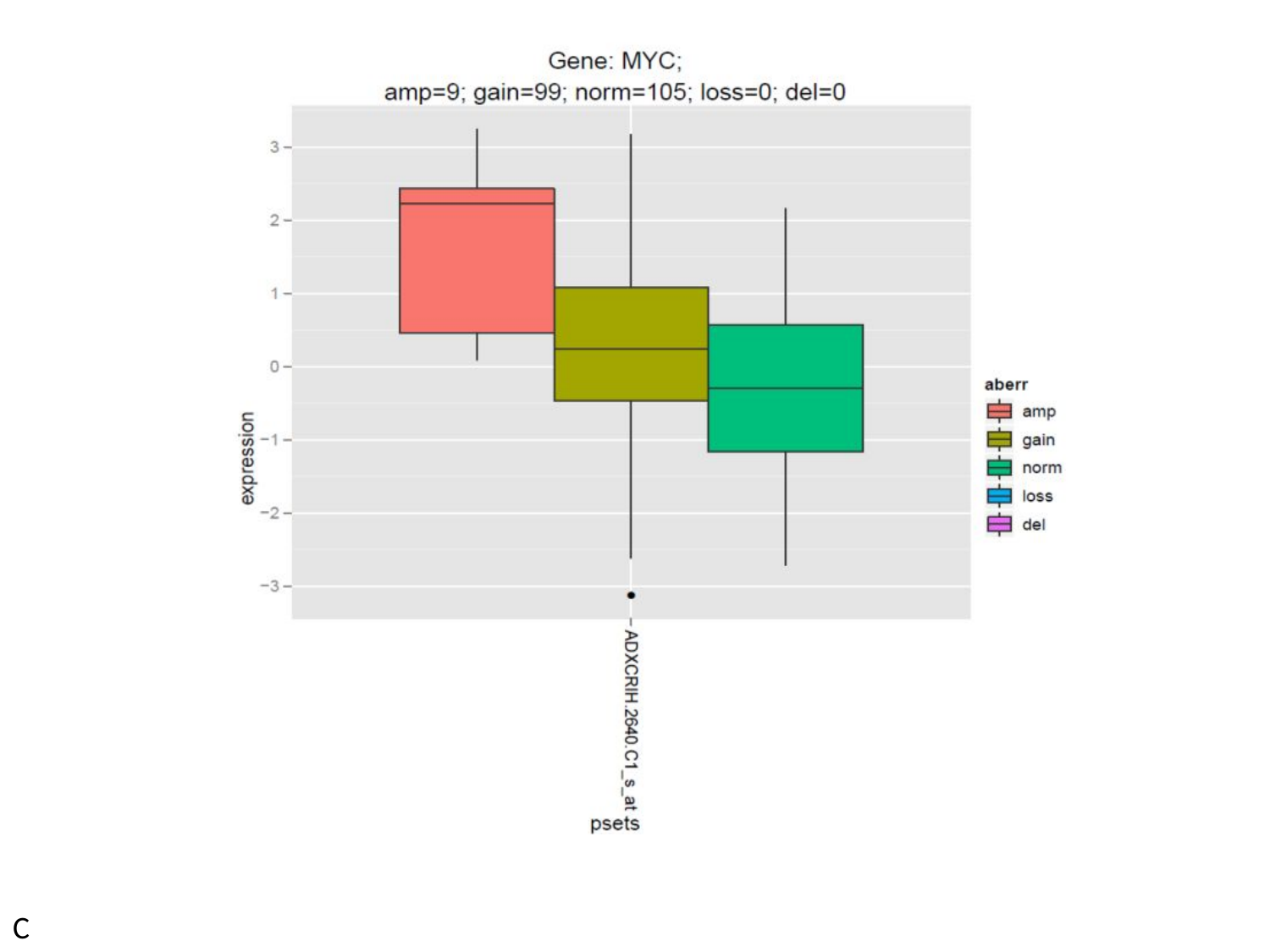

C
