# Supplementary material for: A Comprehensive Characterization of Genome-Wide Copy Number Aberrations in Colorectal Cancer Reveals Novel Oncogenes and Patterns of Alterations
Source: PLoS One. 2012 Jul 31;7(7):e42001. doi: 10.1371/journal.pone.0042001 (PMC3409212; doi:10.1371/journal.pone.0042001)
Supplement: Figure S2 — Kaplan-Meier curves demonstrate CNAs showing significant association with overall survival. (PPT) [file pone.0042001.s002.ppt]

## Slide 1
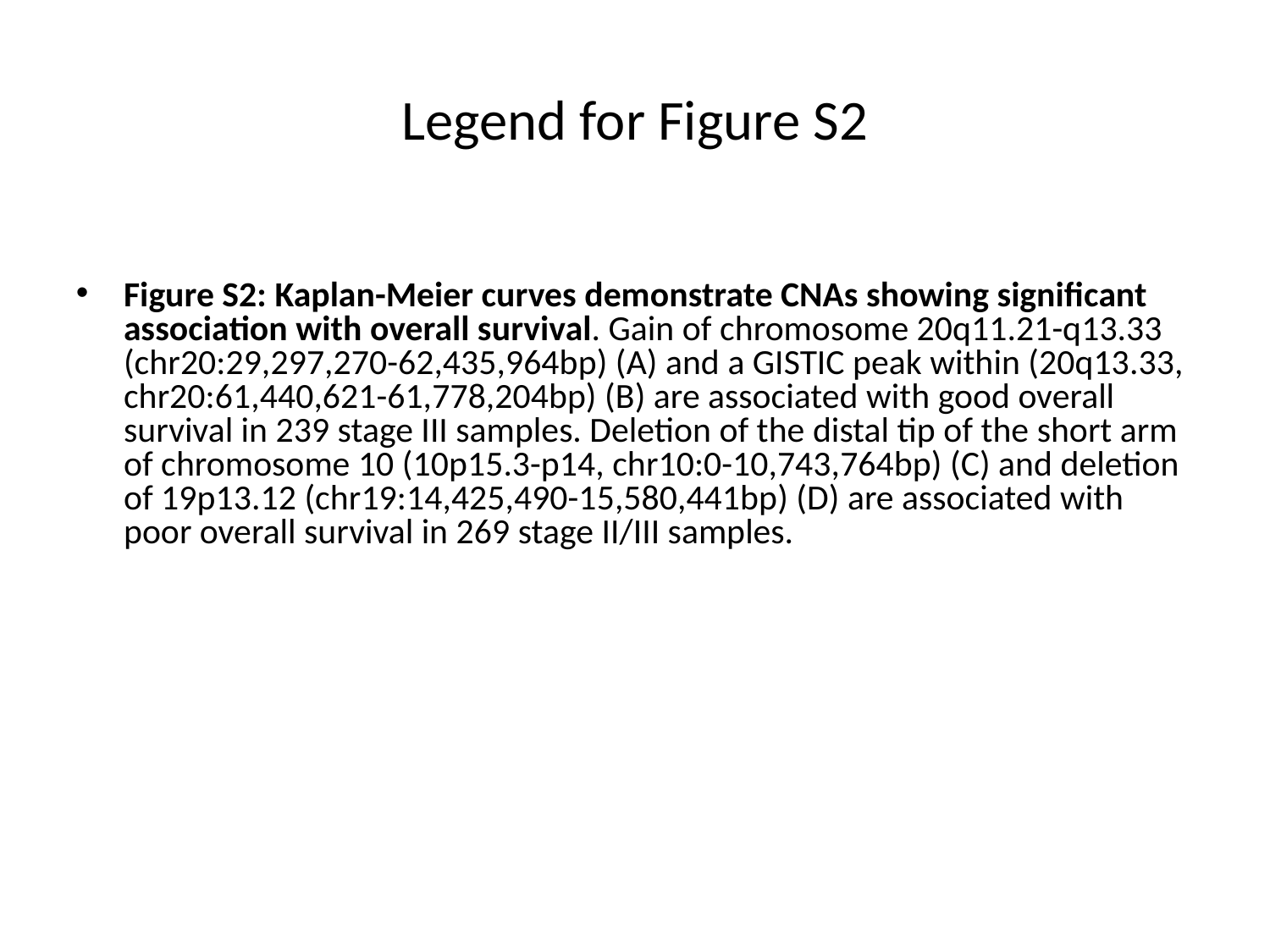

# Legend for Figure S2
Figure S2: Kaplan-Meier curves demonstrate CNAs showing significant association with overall survival. Gain of chromosome 20q11.21-q13.33 (chr20:29,297,270-62,435,964bp) (A) and a GISTIC peak within (20q13.33, chr20:61,440,621-61,778,204bp) (B) are associated with good overall survival in 239 stage III samples. Deletion of the distal tip of the short arm of chromosome 10 (10p15.3-p14, chr10:0-10,743,764bp) (C) and deletion of 19p13.12 (chr19:14,425,490-15,580,441bp) (D) are associated with poor overall survival in 269 stage II/III samples.

## Slide 2
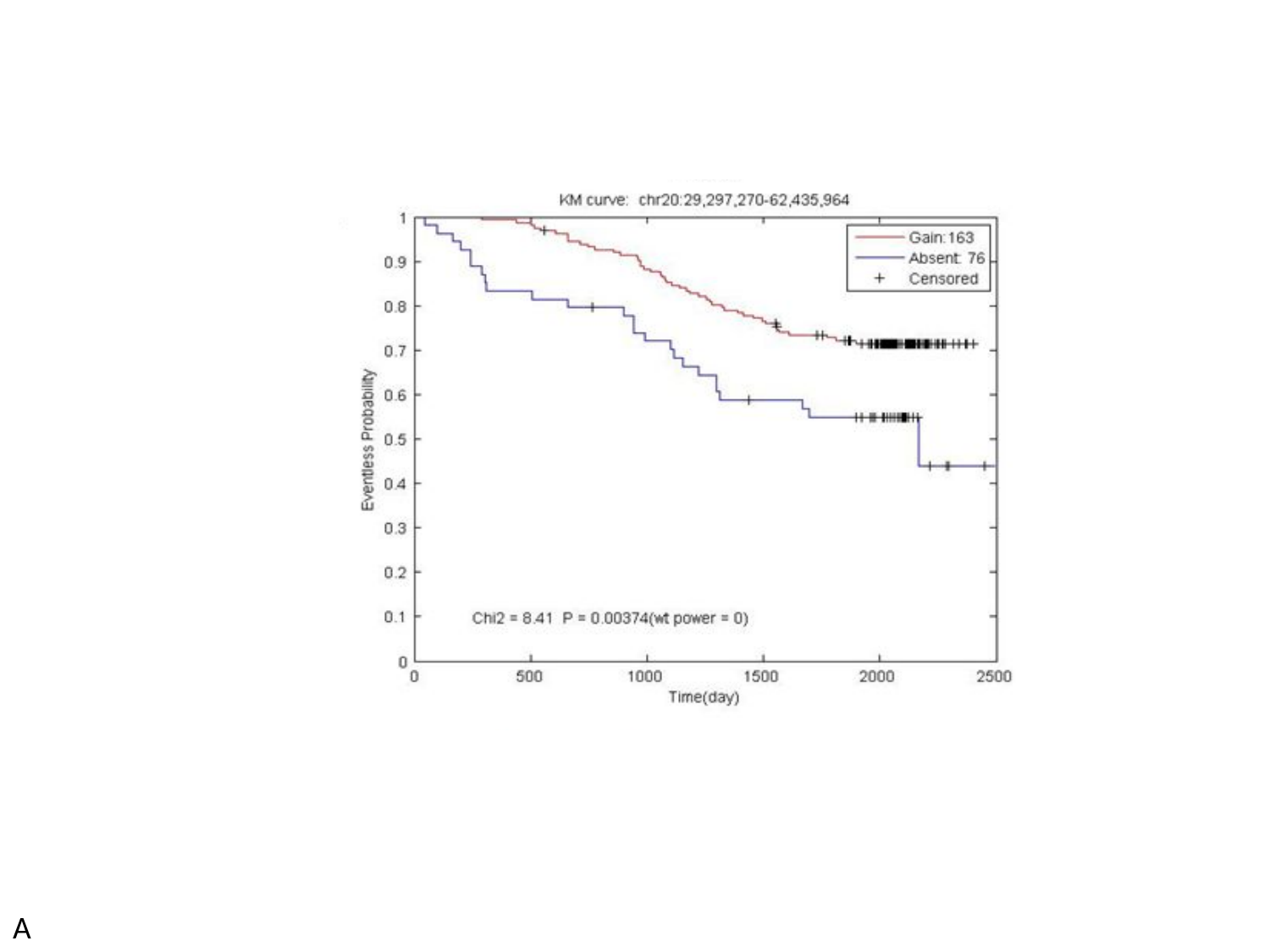

A

## Slide 3
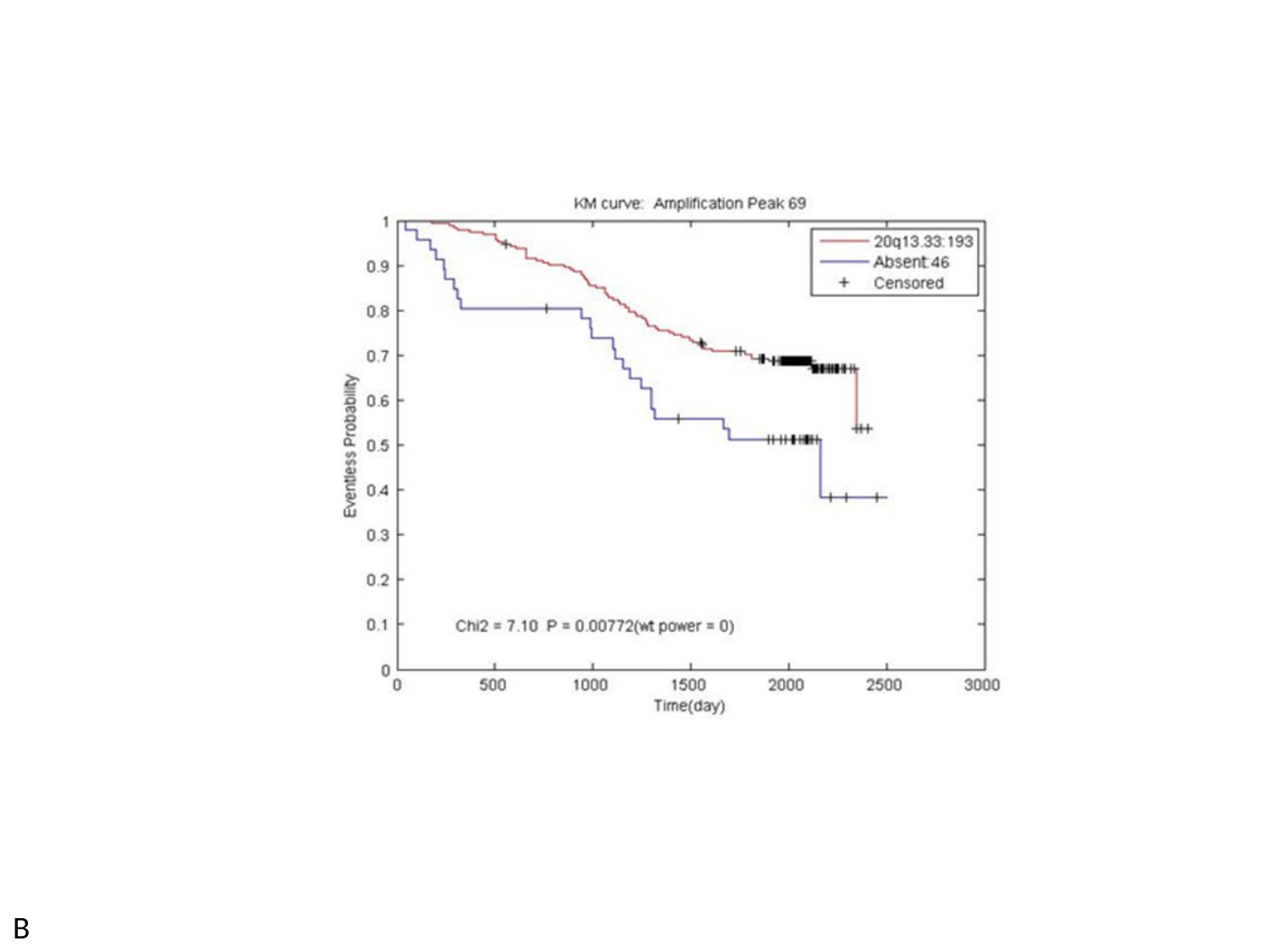

B

## Slide 4
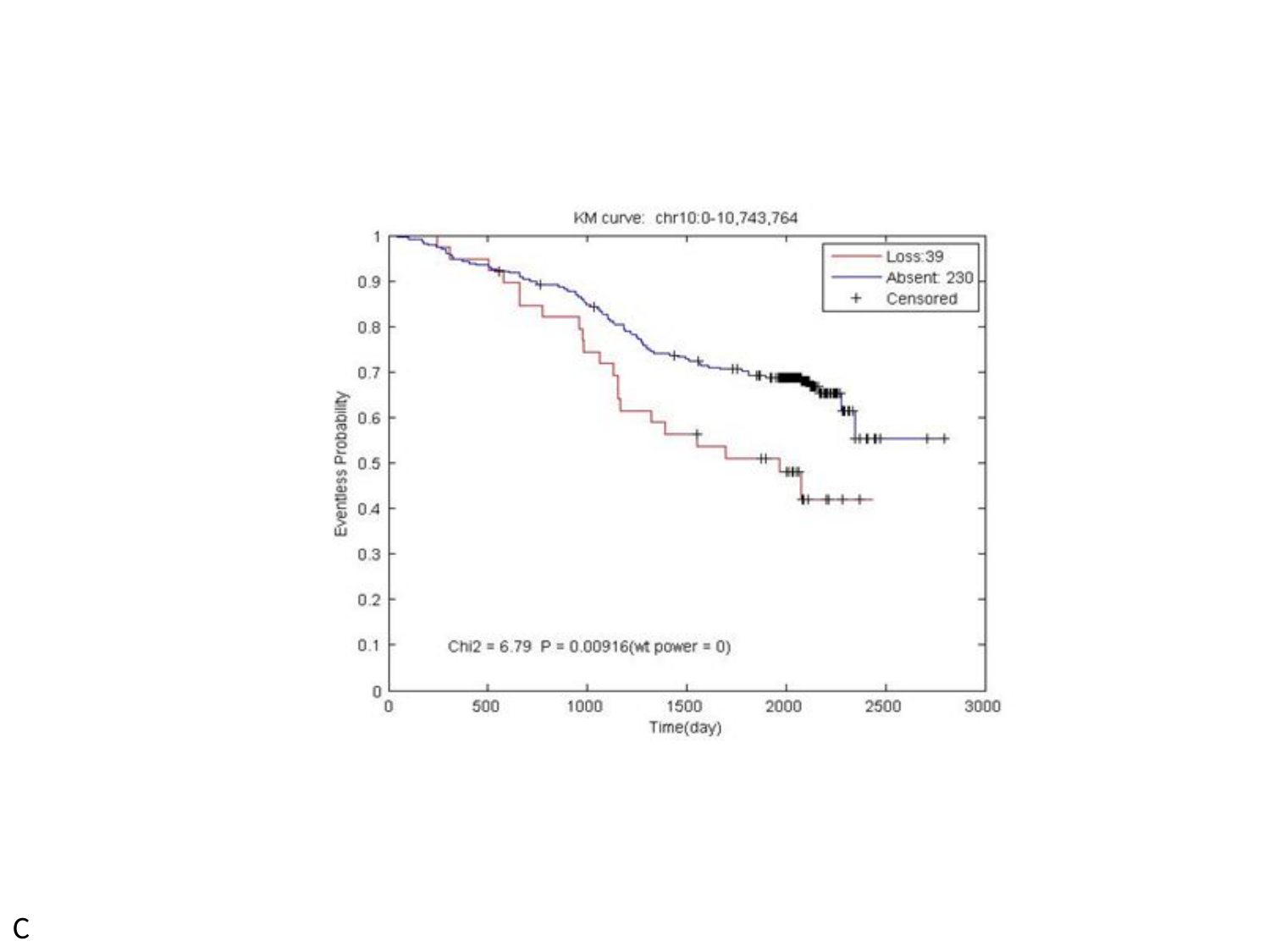

C

## Slide 5
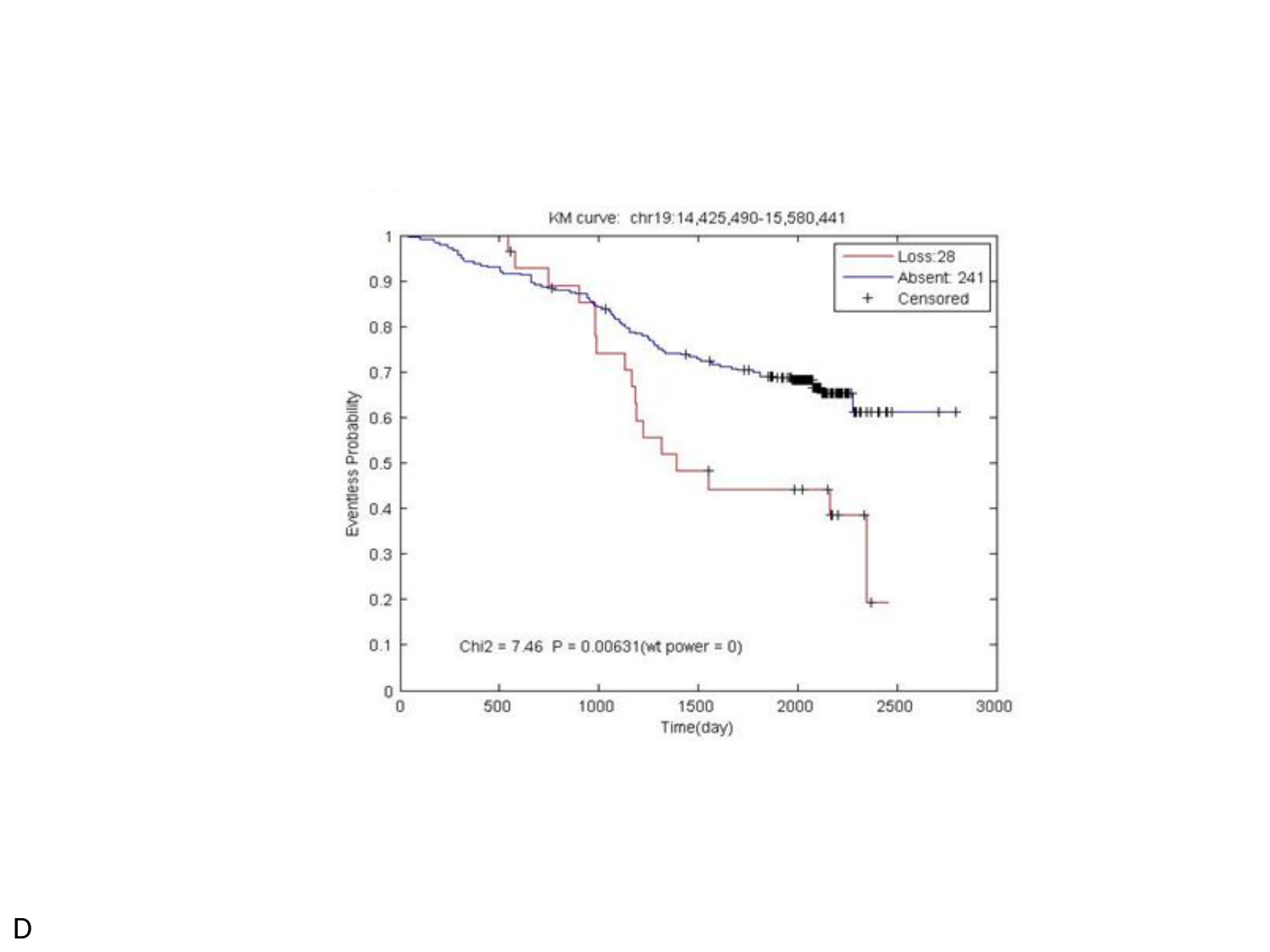

D
